# Tenascin-C Is Increased in Inflammatory Bowel Disease and Is Associated with response to Infliximab Therapy

**DOI:** 10.1155/2019/1475705

**Published:** 2019-11-22

**Authors:** Longui Ning, Sha Li, Jianguo Gao, Liang Ding, Chenhui Wang, Wenguo Chen, Guodong Shan, Fenming Zhang, Jinghua Yu, Guoqiang Xu

**Affiliations:** Department of Gastroenterology, First Affiliated Hospital, Zhejiang University, School of Medicine, Hangzhou 310003, Zhejiang, China

## Abstract

Tenascin-C (TNC) is an extracellular matrix glycoprotein expressed in response to inflammation and tissue damage. The role of TNC in patients with inflammatory bowel disease (IBD) is not well understood. In this study, we analyzed the expression of TNC in the inflamed mucosa of patients with ulcerative colitis (UC) and Crohn's disease (CD). Serum TNC levels were determined by the enzyme-linked immunosorbent assay (ELISA), and the levels of TNC in patients with different disease activities were compared. The expression of TNC was derived from a GEO dataset. THP-1 cells were stimulated with TNC to evaluate the proinflammatory role of TNC. We found higher TNC expression in the inflamed mucosa of patients with UC and CD compared with normal controls (NCs). TNC was mainly expressed in the stromal area of the intestinal mucosa. The median serum levels of TNC were significantly higher in UC (median 74.1 ng/ml, range 42.6–102.1 ng/ml) and CD (median 59.2 ng/ml, range 44.0–80.9 ng/ml). We also found that serum TNC levels were correlated with Mayo scores in UC and Crohn's disease activity index (CDAI) in CD. Through GSE14580, we demonstrated that patients who were nonresponsive to infliximab treatment had higher mucosal TNC mRNA expression. High TNC mRNA expression in the inflamed intestinal mucosa was associated with poor response to infliximab therapy in patients with UC. Furthermore, THP-1 cells stimulated with TNC showed increased expression of IL-6, but not TNF-*α*, IL-8, MCP-1, or IL-1*β*. Thus, increased TNC levels may participate in the pathogenesis of IBD and may serve as a biomarker for disease activity and response to treatment with infliximab.

## 1. Introduction

Inflammatory bowel disease (IBD) describes two conditions: ulcerative colitis (UC) and Crohn's disease (CD). It is a chronic intestinal disorder characterized by relapsing and remitting inflammation across the gastrointestinal tract, mainly the colon and small intestine. Recent epidemiological research has revealed the high incidence of IBD in North America and Europe and in previously low-incidence areas such as Asia [1]. Thus, IBD has become a great economic burden worldwide. Inflammatory cell infiltration and excessive immune responses have been considered the hallmarks of colitis immunopathology.

The extracellular matrix (ECM) serves as a scaffold for cells within tissues and is mainly comprised of three types of proteins: structural proteins, specialized glycoproteins, and proteoglycans. The ECM maintains the structure, organization, and hydration of all tissues and has been shown to play multiple roles in diseases such as osteoarthritis, fibrosis, cancer, and genetic diseases [2, 3]. However, the ECM is a frequently overlooked component in the pathogenesis of IBD. Previous studies have shown that tissues from IBD patients are characterized by an increase in total collagen, fibronectin, and matrix metalloproteinases (MMPs) [4–6], indicating collagen deposition and ECM remodeling. Excessive collagen deposition may lead to stenosis or fibrosis formation. The roles of MMP family molecules in IBD include the regulation of epithelial barrier function, immune response, angiogenesis, fibrosis, and wound healing [7]. Moreover, drugs targeting MMPs have been demonstrated to alleviate intestinal inflammation in animal colitis models [8, 9].

Tenascin-C (TNC) is a hexameric protein comprising an assembly domain, epidermal growth factor-like repeats (EGF-L), fibronectin type III-like repeats (TNIII), and a fibrinogen-like globe (FBG) [10]. It functions as an extracellular matrix glycoprotein and is associated with tissue injury and repair. Upon acute or chronic inflammation, TNC is specifically and transiently upregulated [11–14]. Midwood et al. [15] reported that TNC is an endogenous activator of TLR4-mediated immunity that mediates persistent synovial inflammation and tissue destruction in rheumatoid arthritis (RA). Circulating TNC levels were correlated with bone erosion and acted as a predictor of the effect of infliximab on joint pain in patients with RA [11].

However, the role of TNC in IBD is less well understood. A recent genome-wide association study (GWAS) conducted in African Americans suggested that single-nucleotide polymorphisms (SNPs) in the TNC gene are associated with IBD [16]. A previous study also indicated that serum TNC concentration is higher in patients with CD and UC and is correlated with the course of disease progression [17]. However, the small sample size analyzed in that study limited the reliability of the results [17]. We recently identified that TNC protein levels are significantly higher in inflamed mucosal biopsies from patients with CD and UC, compared with that of healthy controls, through a quantitative proteomic study [18]. In this article, we intend to further clarify the unrecognized role of TNC in IBD.

## 2. Materials and Methods

### 2.1. Subjects

A total of 131 inpatients with IBD at the First Affiliated Hospital of Zhejiang University admitted between September 2016 and December 2018 were included in the present study. There were 40 patients with UC and 91 patients with CD. The study conformed to the ethical guidelines of the Declaration of Helsinki and was approved by the Ethics Committee at First Affiliated Hospital of Zhejiang University. The diagnosis of UC and CD was based on World Gastroenterology Organization Practice Guidelines for the diagnosis and management of IBD [19]. Exclusion criteria included a history of malignancy concurrent with other gastrointestinal diseases and the absence of serum samples taken at baseline. Normal controls (NCs) included 52 healthy individuals without a history of autoimmune diseases or gastrointestinal diseases, who were undergoing routine physical examinations at the Health Management Center of First Affiliated Hospital of Zhejiang University.

For the patients with UC, Mayo scores were used to assess disease activity: scores ≤2 were denoted remission, 3–5 as mild, 6–10 as moderate, and 11-12 as severe. For the patients with CD, BEST CDAI scores were used to assess disease activity: scores <150 were denoted as remission, 150–220 as mild, 220–450 as moderate, and >450 as severe. The Montreal classification was applied to the location and behavior of the disease [20]. The location of UC was categorized as E1 (confined to the rectum), E2 (involved the left colon), and E3 (extensive lesions near the splenic flexure and in the whole colon). The location of CD was categorized as L1 (terminal ileum involvement), L2 (colon involvement), L3 (ileocolon involvement), and L4 (upper GI tract involvement). Disease behavior of CD was categorized into three phenotypes: B1 (nonstricturing and nonpenetrating), B2 (stricturing), and B3 (penetrating). Demographic data were collected from all the included subjects. From each participant, 2 ml of fasting peripheral blood was collected and centrifuged. The supernatants were isolated and then stored at −80°C.

### 2.2. Enzyme-Linked Immunosorbent Assay (ELISA)

Serum TNC levels were determined by the Human Tenascin-C Large (FNIII-0C) kit (Immuno-Biological Laboratories Co., Gunma, Japan). We set up seven solutions of 230 *μ*l of standard TNC solution in a 2 : 1 dilution series of final concentrations between 24 ng/mL and 0.38 ng/mL. Serum samples were diluted 5-fold before use. The standard solutions, blank solution, and diluted serum samples were incubated in a 96-well ELISA plate coated with 100 *μ*l labeled antibody for 30 minutes at 4°C. After washing five times with washing buffer, the plate was incubated with 100 *μ*l chromogen for 30 minutes at room temperature, followed by addition of 100 *μ*l stop solution to each well. Finally, the results were read at a wavelength of 450 nm by an ELISA reader (SpectraMax i3, Molecular Devices, America). The intra- and interassay coefficients of variation as stated by the manufacturers were 3.8% and 3.9% for all assays, respectively.

### 2.3. Immunohistochemical (IHC) Staining

Immunohistochemical staining was performed on paraffin-embedded sections of inflamed intestinal tissues (either under endoscopy or surgery) from patients with UC (*n* = 8) and CD (*n* = 10). NC samples (*n* = 7) were collected from individuals undergoing endoscopic examination. UC and NC tissues were collected from the descending colon, ascending colon, or transverse colon. CD tissues were collected from the ileum, terminal ileum, descending colon, or ascending colon. This study was approved by the Ethics Committee at First Affiliated Hospital of Zhejiang University. Tissues were cut into 5 *μ*m thick sections and stained with H&E before IHC staining. Slides were incubated with primary antibodies against TNC (ab108930, Abcam) at 4°C overnight, followed by incubation with secondary antibody for 1 h at 37°C. The slides were stained with 3,3-diaminobenzidine (DAB) and captured by a light microscope (Leica, Germany). Immunohistochemical staining scores were performed according to a published procedure [21]. In brief, the immunostaining score was evaluated based on the intensity of staining (scored from 0 to 3) and number of stained cells (scored from 0 to 4). The intensity of the staining was graded as 0 (no staining), 1 (weak staining), 2 (moderate staining), and 3 (strong staining). The number of stained cells was graded as 0 (no cell), 1 (<10% of cells), 2 (10–50% of cells), 3 (51–90% of cells), and 4 (>90% of cells). The final score was obtained by multiplying the intensity of staining versus the number of cells.

### 2.4. Analysis of Transcriptomic Data

Transcriptomic data of GSE14580 were downloaded from the Gene Expression Omnibus website (http://www.ncbi.nlm.nih.gov/geo/). Gene expression data of TNC were extracted. In GSE14580, there were 8 patients that belonged to the infliximab responsive group and 16 patients that belonged to the infliximab nonresponsive group. Six control subjects were those who underwent colonoscopy for screening of polyps. The details of experimental design and definition of responsive or nonresponsive of patients in GSE14580 can be referred to the original study [22]. In brief, response was defined as a complete mucosal healing with a Mayo endoscopic score of 0 or 1 and a grade 0 or 1 on the histological score. Patients who did not achieve mucosa healing were considered as nonresponders even if some of them presented endoscopic and/or histological improvement [22]. ROC curve analysis was performed with the gene expression data of TNC, and the optimal cutoff value of TNC expression with the best discriminatory power was determined. Thus, high expression was defined as the expression level more than this cutoff value. Otherwise, it was defined as low expression.

### 2.5. Cell Culture

The human monocyte cell line THP-1 was obtained from ATCC (Rockville, MD). Cells were cultured in RPMI 1640 medium supplemented with 10% fetal bovine serum, 100 U/ml streptomycin, and 100 U/ml penicillin (Gibco; Thermo Fisher Scientific, Inc.) at 37°C and 5% CO_2_. After incubation, the cells were treated with 1 *μ*g/m lipopolysaccharide (LPS, Sigma-Aldrich, St. Louis, MO, USA) or 10 *μ*g/ml recombinant human TNC (CC065; Millipore, Billerica, MA) for 6 h.

### 2.6. RNA Isolation and Real-Time PCR Analysis

Total RNA was extracted from treated THP-1 cells using the Trizol method (Takara, Japan) according to the manufacturer's instructions. The isolated total RNA was reverse transcribed into cDNA using the PrimeScript® RT reagent Kit (Takara, Japan). Quantitative real-time PCR was performed using the ABI 7500 real-time PCR System (Applied Biosystems, Thermo Fisher Scientific, USA) with the SYBR Green reagent (Takara, Japan). The relative expression levels of the target genes were normalized to GAPDH and calculated using the 2^−∆∆Cq^ method. Forward and reverse sequences of the primers used for all target genes and GAPDH are listed in Supplementary [Supplementary-material supplementary-material-1].

### 2.7. Statistical Analysis

Normally distributed variables are expressed as mean ± standard deviation (SD), and nonnormally distributed variables are expressed as median (interquartile range, 25th–75th percentiles) when appropriate. Numbers and percentages are used to represent categorical variables. Dichotomous variables were analyzed by the chi-square test. One-way analysis of variance (ANOVA) was utilized for normally distributed continuous variables among three or more groups. The Kruskal–Wallis test was used for comparisons of nonnormally distributed continuous variables with more than two groups. Correlations were determined using Spearman's coefficient to determine the association between TNC levels and the CDAI or Mayo scores. GraphPad Prism 6 (GraphPad Prism Software Inc., San Diego, CA, USA) and SPSS version 22.0 (SPSS Inc., Chicago, IL, USA) were used for statistical analysis. A two-sided *P* value < 0.05 was considered statistically significant.

## 3. Results

### 3.1. Confirmation of TNC Protein Expression in IBD

**Figure 1:**
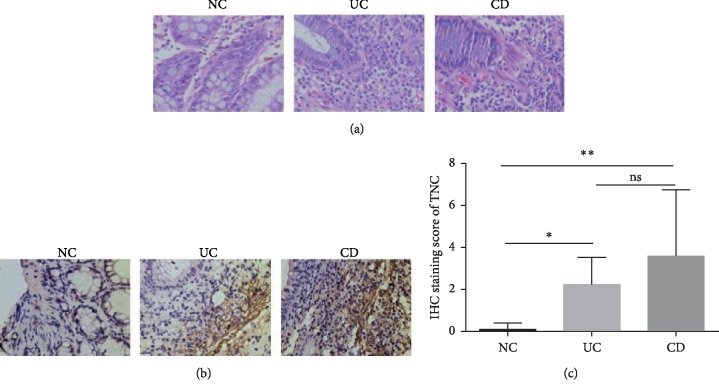
Increased expression of TNC in intestine of patients with UC and CD. (a) Representative H&E images of NCs (*n* = 7), UC (*n* = 8), and CD (*n* = 10) (original magnification, ×400). (b) Representative IHC images of TNC from NCs (*n* = 7), UC (*n* = 8), and CD (*n* = 10) (distal colon) (original magnification, ×400). (c) Comparison of IHC staining score among groups. ^*∗*^*P* < 0.05 and ^*∗∗*^*P* < 0.01. UC, ulcerative colitis; CD, Crohn's disease; NCs, normal controls.

We previously identified that TNC protein levels were higher in the inflamed mucosa from patients with CD and UC compared to that of healthy controls through a quantitative proteomic research [18]. In this study, we used IHC to confirm the protein expression of TNC. As seen in Figures 1(a) and 1(b), the expression of TNC was significantly higher in inflamed mucosa of UC and CD (Figure 1(c)). Furthermore, TNC was mainly expressed in the lamina propria of the intestinal mucosa.

### 3.2. Serum TNC Levels Are Increased in IBD

**Figure 2:**
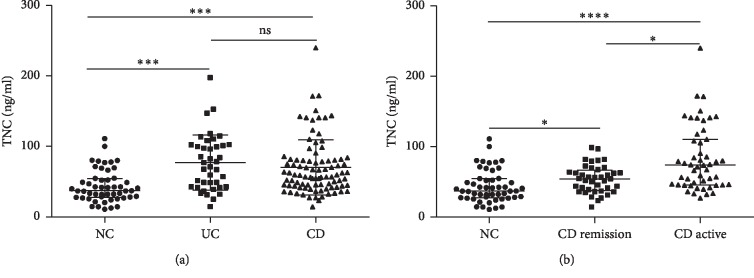
Serum TNC levels are increased in IBD. (a) Serum TNC levels in patients with UC (*n* = 40), CD (*n* = 91), and NCs (*n* = 52). (b) Serum TNC levels in NCs (*n* = 52), CD remission (*n* = 40), and CD active (*n* = 51). ^*∗*^*P* < 0.05, ^*∗∗∗*^*P* < 0.001, and ^*∗∗∗∗*^*P* < 0.0001. UC, ulcerative colitis; CD, Crohn's disease; NCs, normal controls.

The baseline characteristics of included subjects are shown in Table 1. There were 52 NCs, 40 UC, and 91 CD patients. No differences were observed in age and gender among the three groups. The median serum levels of TNC were significantly higher in UC (median 74.1 ng/ml, range 42.6–102.1 ng/ml) and CD (median 59.2 ng/ml, range 44.0–80.9 ng/ml) compared with NC (median 37.1 ng/ml, range 27.7–54.6 ng/ml) (Figure 2(a)). There was no difference between patients with UC and CD. The classification of stage and group of IBD is shown in Table 2. Since all the patients with UC are in the active stage, we compared serum TNC levels of CD in the active stage and in remission. The level of TNC was significantly higher in CD in the active stage and in remission compared with that of the controls (Figure 2(b)). The level of TNC was also higher in CD in the active stage than that of the remission stage (Figure 2(b)).

Based on the Montreal classification, serum TNC level in CD and UC was further analyzed according to the age at diagnosis, disease location, disease behavior, and disease extension. Although there was trend toward higher TNC levels in UC patients with a wider disease extension and in CD patients with colon involvement and a penetrating behavior, no significant differences were found amongst patients (Table 3).

**Figure 3:**
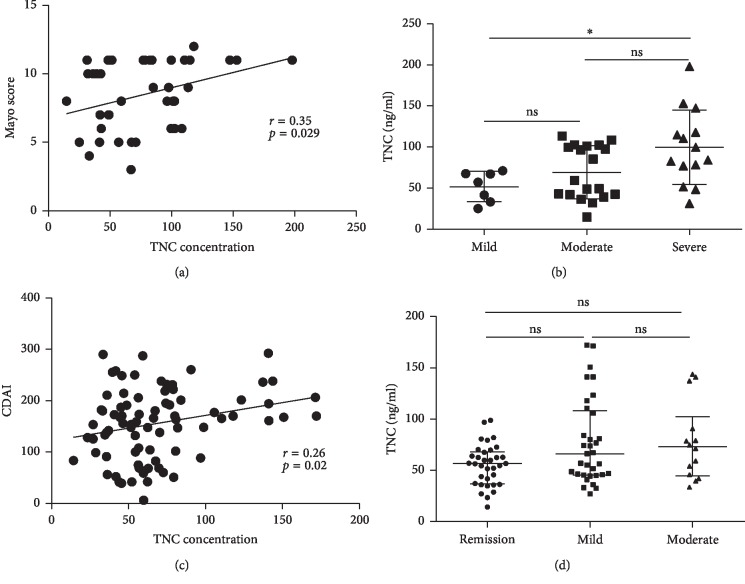
Serum TNC levels and severity of IBD. (a) Correlation between TNC levels and Mayo score in patients with UC; (b) serum TNC levels according to disease severity in patients with UC; (c) correlation between TNC levels and CDAI in patients with CD; (d) serum TNC levels according to disease severity in patients with CD. ^*∗*^*P* < 0.05. UC, ulcerative colitis; CD, Crohn's disease.

Serum TNC levels were correlated with Mayo scores in UC (Figure 3(a)) and CDAI scores in CD (Figure 3(c)). In patients with UC, there was an observable trend toward higher TNC levels according to the severity of disease, with the highest TNC levels found in patients with severe disease (Figure 3(b)). Although there was a trend toward higher TNC levels according to the severity of disease in CD patients, the difference was not statistically significant (Figure 3(d)).

### 3.3. Mucosal TNC mRNA Expression is Associated with Infliximab Therapy in UC

**Figure 4:**
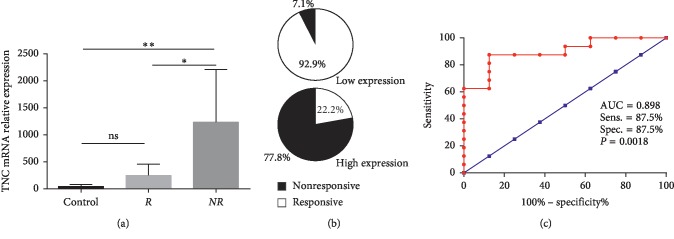
Mucosa TNC mRNA levels are associated with response to infliximab therapy in UC patients. (a) mRNA levels of TNC in control, responsive (R), and nonresponsive (NR) group; (b) rates of responsive or nonresponsive to infliximab in patients with low or high TNC expression; (c) receiver operating characteristic (ROC) curves illustrating the specificity and sensitivity in mucosa TNC levels to differentiate infliximab responsive or nonresponsive for UC. ^*∗*^*P* < 0.05 and ^*∗∗*^*P* < 0.01.

Arijs et al. investigated the gene signature for predicting response to infliximab treatment in UC patients [22]. They identified several genes that separated responders from nonresponders with high accuracy. By downloading the transcriptomic data from their study in the GEO dataset, we found that TNC mRNA levels were significantly higher in infliximab nonresponsive (NR) patients than in the infliximab responsive (R) patients and controls (Figure 4(a)). Rates of infliximab response or nonresponse in these patients are shown in Figure 4(b). Complete mucosal healing following infliximab therapy was achieved by 92.9% of patients with low TNC expression, whereas only 22.2% in those with high TNC expression. Notably, expression of TNC at baseline was strongly associated with poor primary response to infliximab in this cohort of UC patients (Figure 4(c)).

### 3.4. TNC Promotes IL-6 Expression in Human Monocytes

**Figure 5:**
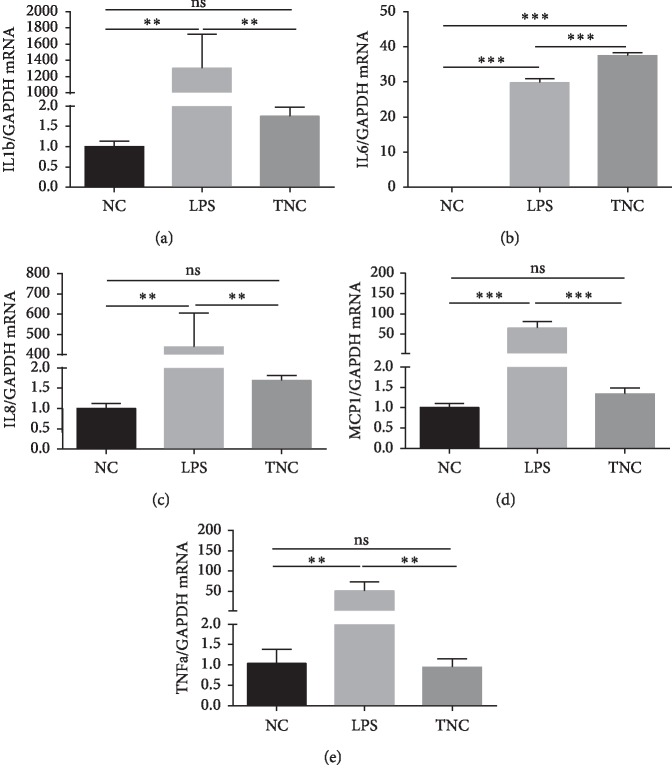
TNC promotes IL-6 expression in THP-1 cells. THP-1 cells were stimulated with LPS or TNC for 6 hours. mRNA expression of IL-1*β* (a), IL-6 (b), IL-8 (c), MCP-1 (d), and TNF-*α* (e) was assessed in the three groups (*n* = 3/group). ^*∗∗*^*P* < 0.01 and ^*∗∗∗*^*P* < 0.001.

To elucidate whether TNC can directly affect inflammatory cytokines expression in immune cells, we investigated inflammatory cytokine expression in TNC-stimulated THP-1 cells, a human monocyte/macrophage cell line. We found that LPS increased the gene expression of IL-6, TNF-*α*, IL-8, MCP-1, and IL-1*β*, whereas TNC stimulation only increased the expression of IL-6 (Figure 5). These results suggested that TNC may specifically promote IL-6 expression in THP-1 cells.

## 4. Discussion

In the present study, we demonstrated increased mucosal and serum TNC expression in patients with IBD, which was consistent with our previous proteomic results [18]. Serum TNC levels were correlated with Mayo and CDAI scores. By using transcriptomic data from the GEO dataset, we also demonstrated that high TNC expression in the inflamed intestinal mucosa was associated with poor response to infliximab therapy in patients with UC. Thus, mucosal TNC mRNA expression may be used as a predictor of response to infliximab.

We found that there was no significant difference in TNC levels among CD patients with different behaviors, which is different from results in Erdem's group [23], who indicated that CD patients with an obstructive behavior (B2) had higher circulating TNC levels than the inflammatory and fistulizing groups. Compared with our study, the number of CD was smaller and the proportion of patients with B2 was higher in Erdem's study (37.2% and 17.6%, respectively). Therefore, further studies with a larger sample size are needed to clarify these contradicting findings. In our study, serum TNC levels are not correlated with disease activity in CD, and this was opposed to the results found in Riedl's study [17]. As in our study, there were few cases with severe activity and a small number of cases with moderate activity, which may result in the lack of significance.

Little or no TNC expression is found in most healthy human tissues. However, it is upregulated at sites of tissue injury and inflammation in a variety of diseases such as RA [11], systemic lupus erythematosus (SLE) [12], chronic renal disease [24], and ischemic heart disease [25]. Therefore, TNC is not disease-specific, but rather a universal and ubiquitous marker of tissue injury. We found increased TNC expression in the serum and in the inflamed mucosa of patients with IBD. Serum TNC levels were positively correlated with disease activity in both UC and CD. It is unclear whether elevated TNC levels are the cause or consequence in tissue damage and inflammation. Goh et al. showed that primary human monocytes, MDMs, and MDDCs exhibited significantly increased expression of TNC mRNA in response to stimulation with LPS [26]. TNC synthesis is transcriptionally regulated and requires the specific activation of AKT/PI3K and NF-*κ*B signaling pathways. On the other hand, TNC acts as an endogenous activator of innate immunity by promoting the synthesis of inflammatory cytokines via activation of TLR4 [15]. This would form an autocrine feedback loop where expression of TNC is induced upon activation of cell surface TLRs, and subsequent activation of TLR4 by TNC promotes the synthesis of proinflammatory cytokines [26].

The role of TNC in the pathogenesis of IBD is currently unclear. A study using an animal model suggested that mucosal TNC is produced by subepithelial myofibroblasts in animal colitis models [27]. TNC functions to promote intestinal epithelial cell (IEC) migration and wound healing, contributing to intestinal mucosal protection. However, the role of TNC in intestinal immunity has not been investigated. A previous study revealed that TNC could activate peritoneal macrophages via the integrin *α*V*β*3/NF–*κ*B/IL-6 axis [28]. Blocking *β*3 integrin by an anti-*β*3 integrin neutralizing antibody reversed the effects of TNC in activating macrophages. In primary human macrophages and synovial membrane cells, TNC promoted proinflammatory cytokine (IL-6 and TNF-*α*) synthesis, mainly mediated by the fibrinogen-like globe (FBG) domain [15]. In TLR4−/− or MyD88−/− microphages and embryonic fibroblast, FBG failed to stimulate cytokine synthesis, indicating that FBG-mediated cytokine synthesis is MyD88 and TLR4 dependent [15]. In our study, we noticed that TNC-stimulated THP-1 cells exhibited increased expression of IL-6, but not TNF-*α*, IL-1*β*, or MCP-1, which is not consistent with other studies that used primary macrophages [15, 28, 29]. This difference may be due to the different recombinant TNCs or cell lines used. Altogether, it is interesting to investigate whether TNC plays a proinflammatory role in intestinal immunity in the context of IBD in the future.

We also found that high expression of TNC mRNA in the mucosa of UC is associated with poor infliximab response. A previous GWAS suggested that SNPs in the TNC gene are associated with IBD [16]. However, it is uncertain whether SNPs in the TNC gene are related with TNC expression. We assume that TNC, as a proinflammatory factor, may play a TNF-*α* independent role, which may explain why patients with high TNC expression respond poorly to anti-TNF-*α* therapy. In Magnusson's study [30], they revealed that there was a marked decrease in levels of TNC both at week 2 and week 14 for therapy responders. In contrast, no changes in serum levels of TNC were detected for nonresponders. Therefore, they demonstrated serum TNC levels are affected by infliximab therapy. However, whether serum TNC levels can be used as a marker to infliximab therapy response is unclear, which is an interesting focus for future research.

Our study has several limitations. Firstly, the number of patients with UC was relatively small. All the included UC patients were in the active stage, while most of patients with CD (44%) were in remission, which may lead to bias in the results of subgroup analyses. Furthermore, we did not detect changes in TNC levels before and after treatment. Although we revealed that mucosal TNC mRNA expression may act as a predictor of infliximab response, we did not determine whether serum TNC levels are associated with infliximab response, as serum markers are more suitable as noninvasive markers than histological markers. Therefore, our future research will focus on studying whether serum TNC levels are related to infliximab therapy response.

## 5. Conclusion

In conclusion, we revealed higher TNC levels in the serum and mucosal tissues in patients with UC and CD. Serum TNC levels were correlated with disease activity in UC and CD. Finally, high TNC mRNA expression in the inflamed mucosa was associated with poor response to infliximab therapy. Thus, TNC may be involved in intestinal immunity and may serve as a useful biomarker for IBD.

## Figures and Tables

**Table 1:** Characteristics of included subjects.

	NC	UC	CD	*P* value
Age median (years)	39.0 (33.0, 46.0)	38.0 (31.3, 53.0)	35.0 (24.5, 50.5)	0.357
Gender, M/F	30/21	26/14	53/38	0.755
TNC median (ng/ml)	37.1 (27.7, 54.6)	74.1 (42.6, 102.1)	59.2 (44.0, 80.9)	<0.001

NCs, normal controls; UC, ulcerative colitis; CD, Crohn's disease.

**Table 2:** Stage and group of IBD.

Disease	Remission stage	Active stage			Total
		Mild	Moderate	Severe	
UC	0	7	19	14	40
CD	40	35	16	0	91
IBD	40	42	35	14	131

UC, ulcerative colitis; CD, Crohn's disease; IBD, inflammatory bowel disease.

**Table 3:** Relationship between Montreal classification and serum TNC levels.

	Number	Serum TNC (ng/L)	*P* value
CD			
Age at diagnosis			0.579
A1 (≤16)	4	44.9 (44.3, 60.5)	
A2 (17–20)	54	57.0 (43.9, 79.1)	
A3(>40)	33	61.2 (45.6, 89.0)	
Location			0.916
L1	22	55.1 (48.8, 79.4)	
L2	30	65.6 (42.5, 89.0)	
L3	39	57.0 (44.7, 78.4)	
L4	0	−	
Disease behavior			0.278
B1	51	57.0 (45.0, 81.2)	
B2	16	56.4 (38.6, 70.9)	
B3	24	68.5 (46.1, 87.8)	

UC			
Disease extension			0.729
E1	3	59.3 (54.2, 83.8)	
E2	13	71.1 (33.3, 102.4)	
E3	24	77.8 (48.8, 100.1)	

CD, Crohn's disease; UC, ulcerative colitis.

## Data Availability

Publically available transcriptomic data used in this study are available at the NCBI Gene Expression Omnibus under the accession code GSE14580.

## Supplementary Materials

Supplementary Materials Supplementary Table 1. The sequences for qPCR primers.
