# Supplementary material for: Tenascin-C Is Increased in Inflammatory Bowel Disease and Is Associated with response to Infliximab Therapy
Source: Biomed Res Int. 2019 Nov 22;2019:1475705. doi: 10.1155/2019/1475705 (PMC6893280; doi:10.1155/2019/1475705)
Supplement: Supplementary Materials — Supplementary Table 1. The sequences for qPCR primers. [file 1475705.f1.docx]

| Gene | Forward (5’-3’ sequence) | Reverse (5’-3’ sequence) |
| --- | --- | --- |
| Human-IL-1β | ATGATGGCTTATTACAGTGGCAA | GTCGGAGATTCGTAGCTGGA |
| Human-TNF-α | GAGGCCAAGCCCTGGTATG | CGGGCCGATTGATCTCAGC |
| Human-IL-6 | CCTGAACCTTCCAAAGATGGC | TTCACCAGGCAAGTCTCCTCA |
| Human-MCP-1 | CAGCCAGATGCAATCAATGCC | TGGAATCCTGAACCCACTTCT |
| Human-IL-8 | ACTGAGAGTGATTGAGAGTGGAC | AACCCTCTGCACCCAGTTTTC |
| Human-GAPDH | ACAACTTTGGTATCGTGGAAGG | GCCATCACGCCACAGTTTC |

Supplementary table 1 The sequences for qPCR primers
